# Triceps Surae Muscle Architecture Adaptations to Eccentric Training

**DOI:** 10.3389/fphys.2019.01456

**Published:** 2019-11-26

**Authors:** Jeam Marcel Geremia, Bruno Manfredini Baroni, Rodrigo Rico Bini, Fabio Juner Lanferdini, Amanda Rodrigues de Lima, Walter Herzog, Marco Aurélio Vaz

**Affiliations:** ^1^Laboratório de Pesquisa do Exercício, Escola de Educação Física, Fisioterapia e Dança, Universidade Federal do Rio Grande do Sul, Porto Alegre, Brazil; ^2^Departamento de Fisioterapia, Universidade Federal de Ciências da Saúde de Porto Alegre, Porto Alegre, Brazil; ^3^Holsworth Research Initiative, La Trobe Rural Health School, La Trobe University, Bendigo, VIC, Australia; ^4^Laboratório de Biomecânica, Centro de Desportos, Universidade Federal de Santa Catarina, Florianópolis, Brazil; ^5^Faculty of Kinesiology, Engineering, Medicine and Veterinary Medicine, University of Calgary, Calgary, AB, Canada

**Keywords:** eccentric exercise, muscle architecture, muscle plasticity, triceps surae, ultrasonography

## Abstract

**Background:**

Eccentric exercises have been used in physical training, injury prevention, and rehabilitation programs. The systematic use of eccentric training promotes specific morphological adaptations on skeletal muscles. However, synergistic muscles, such as the triceps surae components, might display different structural adaptations due to differences in architecture, function, and load sharing. Therefore, the purpose of this study was to determine the effects of an eccentric training program on the triceps surae (GM, gastrocnemius medialis; GL, gastrocnemius lateralis; and SO, soleus) muscle architecture.

**Methods:**

Twenty healthy male subjects (26 ± 4 years) underwent a 4-week control period followed by a 12-week eccentric training program. Muscle architecture [fascicle length (FL), pennation angle (PA), and muscle thickness (MT)] of GM, GL, and SO was evaluated every 4 weeks by ultrasonography.

**Results:**

Fascicle lengths (GM: 13.2%; GL: 8.8%; SO: 21%) and MT (GM: 14.9%; GL: 15.3%; SO: 19.1%) increased from pre- to post-training, whereas PAs remained similar. GM and SO FL and MT increased up to the 8th training week, whereas GL FL increased up to the 4th week. SO displayed the highest, and GL the smallest gains in FL post-training.

**Conclusion:**

All three synergistic plantar flexor muscles increased FL and MT with eccentric training. MT increased similarly among the synergistic muscles, while the muscle with the shortest FL at baseline (SO) showed the greatest increase in FL.

## Introduction

Muscle architecture (i.e., the geometrical arrangement by which muscle fibers are organized with respect to a muscle’s line of action) has an important role in skeletal muscle force production (Fukunaga et al., 1997; Lieber and Fridén, 2000). Fascicle length (FL) is associated with the serial sarcomere number, which has a direct impact on the fiber/muscle shortening velocity and excursion (Edman et al., 1985; Lieber and Fridén, 2000; Blazevich, 2006). Longer FL is associated with improved performance in activities demanding high velocities of shortening (Abe et al., 2000, 2001; Kumagai et al., 2000), whereas a reduction in FL with aging explains a large part of the reduced maximal shortening velocity in older compared to young adults (Thom et al., 2007). Muscle fiber pennation angle (PA) is related to the parallel number of sarcomeres within a fiber, and is related to the fiber diameter and its maximal capacity for force production (Fukunaga et al., 1997; Blazevich and Sharp, 2005). Hypertrophied muscles (i.e., with a large number of sarcomeres in parallel) tend to have high PA (Kawakami et al., 1993), while muscles of elderly (Narici et al., 2003) or disuse atrophied muscles (Suetta et al., 2009) (i.e., with a small number of parallel sarcomeres), tend to have small PA. Muscle thickness (MT) is influenced by both the serial and parallel sarcomere number, as it depends on FL and PA (Fukunaga et al., 1997; Baroni et al., 2013b).

Structural changes accompanying (or due to) strength training provide insights into the muscle’s ability to accommodate architecture adaptations specific to the movement’s mechanical demands. Although traditional isoinertial resistance training is executed with a constant external load in the concentric and eccentric phases of movement (Walker et al., 2016), training programs often involve isometric contractions (Kitai and Sale, 1989; Oranchuk et al., 2019) or have an emphasis on the concentric (Blazevich et al., 2007; Timmins et al., 2016b) or eccentric (Baroni et al., 2013b; Geremia et al., 2018b) phases. Evidence suggests that training programs neglecting eccentric actions do not prepare subjects for the eccentric demands encountered in sports and activities of daily living (Barstow et al., 2003; LaStayo et al., 2003; Lovering and Brooks, 2014; Franchi et al., 2017). Therefore, strength training with an emphasis on eccentric movement execution has become popular for the purpose of injury prevention (LaStayo et al., 2003; Goode et al., 2015) and rehabilitation (Rees et al., 2008; Murtaugh and Ihm, 2013; Frizziero et al., 2014), as well as physical fitness improvement in healthy subjects (Baroni et al., 2013c; Geremia et al., 2018a, b).

Several studies demonstrated changes in knee extensor muscle architecture with eccentric training (Higbie et al., 1996; Hortobágyi et al., 1996; Housh et al., 1996; Seger et al., 1998; Reeves et al., 2003; Blazevich et al., 2007; Santos et al., 2010; Raj et al., 2012; Baroni et al., 2013b, c, 2015). Most of these studies reported an increase in FL (Blazevich and Sharp, 2005; Blazevich et al., 2007; Baroni et al., 2013b), while results on changes in PA were mixed (Blazevich et al., 2007; Reeves et al., 2009; Raj et al., 2012; Baroni et al., 2013b). Eccentric training has been found to produce significant changes in muscle architecture after training periods as short as 4 weeks (Baroni et al., 2013b, c; Geremia et al., 2018b). However, different muscle groups (Mulder et al., 2009) or even muscles within the same functional group (Butterfield et al., 2005), may adapt differently when exposed to the same eccentric training stimulus.

Eccentric training programs for the triceps surae muscle have been widely used in tendon injury rehabilitation (Alfredson et al., 1998; Kingma et al., 2007; Woodley et al., 2007), as well as to improve performance in healthy subjects (Geremia et al., 2018a, b). However, findings on the effects of eccentric training on the triceps surae muscles are conflicting. While some studies show that eccentric training promotes an increase in FL, PA, and MT (Duclay et al., 2009; Geremia et al., 2018b), other studies did not find changes in these architectural outcomes (Raj et al., 2012; Fouré et al., 2013). In the majority of studies (Duclay et al., 2009; Raj et al., 2012), it was assumed that adaptations observed for the gastrocnemius medialis (GM) were representative of the entire triceps surae muscle group. However, different muscle architecture adaptations were observed after 6 weeks of stretching between the GM and the gastrocnemius lateralis (GL) (Simpson et al., 2017). This may be due to the fact that load sharing between the synergistic muscles is not homogeneous, and different mechanical loads have been observed for the different components of the triceps surae muscle (Crouzier et al., 2018). Also, differential adaptations to eccentric training have been observed for the vastus lateralis and vastus intermedius of rats exposed to a chronic downhill training protocol (Butterfield et al., 2005). Furthermore, short muscle fibers are more susceptible to muscle damage caused by eccentric training than long muscle fibers (Proske and Morgan, 2001; Baroni et al., 2013b). Considering the differences in muscle fascicular geometry of the triceps surae components (GM, GL, and SO, soleus) (Wickiewicz et al., 1983; Maganaris et al., 1998; Fouré et al., 2013), eccentric exercise may cause different levels of micro-damage in these muscles. Therefore, the idea of a specific adaptive response for the different components of the triceps surae muscle seems reasonable.

We have previously demonstrated time-dependent architectural adaptations in human knee extensor muscles (Baroni et al., 2013b). However, we were unable to find studies evaluating time-dependent adaptations in muscle architecture for the three triceps surae muscles. Considering the conflicting results regarding eccentric training adaptations of the triceps surae muscles, the limited information on architectural adaptations of the individual triceps surae muscles, and the lack of longitudinal, time-dependent adaptations of muscles subjected to eccentric training programs, the purpose of this study was to determine the effects of a 12-week eccentric training program on the architecture of the GM, GL, and SO muscles. Based on the literature (Proske and Morgan, 2001; Duclay et al., 2009; Baroni et al., 2013b; Geremia et al., 2018b), we hypothesize that eccentric training will promote adaptations in muscle architecture, causing larger increases in FL and MT in the muscle with the shortest FL (i.e., SO). In addition, we also expected that these adaptations will occur between 4 and 8 weeks of the training program.

## Materials and Methods

### Participants

All procedures in this study were approved by the Ethics Research Committee of the Universidade Federal do Rio Grande do Sul (Protocol number: 787.347; CAAE: 32907414.9.0000.5347). All participants signed an informed consent form prior to their participation. Healthy and physically active male subjects (18–35 years of age) were invited to participate in the study. Participants were excluded if (1) they were enrolled in any lower limb strength training program within 6 months of this study; (2) they had any musculoskeletal injury of the lower and/or the upper limbs; (3) they had any contra-indications for maximal effort contractions (cardiovascular, musculoskeletal, respiratory, or neurologic diseases); (4) they had any difficulty in understanding and/or executing the testing and training protocols at the isokinetic dynamometer; or if (5) they missed two or more of the training sessions.

The G^∗^Power software (Kiel University, Germany) was used to calculate the sample size of 15 subjects using an effect size (ES) of 0.30, a significance level of 0.05; and a power of 0.80 (Rhea, 2004; Faul et al., 2007; Baroni et al., 2013c; Geremia et al., 2018b). Twenty-four participants started the training program. One participant abandoned the study for personal reasons, and three participants were excluded due to ankle pain during the training program. Therefore, 20 participants (university students; 26 ± 4 years; 1.75 ± 0.08 m height; 75 ± 9 kg of body mass; 24 ± 2 kg/m^2^ of body mass index), physically active men, completed the eccentric training program. Fourteen participants completed all 23 training sessions, and six participants completed 22 training sessions (adherence: 98.8%).

### Experimental Design

A longitudinal trial was designed to determine morphological adaptations of GM, GL, and SO during the 12-week plantar flexor eccentric training program. Triceps surae muscle architecture was evaluated five times: at the start (Baseline); after a 4-week control period (Pre-training), in which participants were instructed not to change their regular physical exercise regimen; after 4 (Post-4); after 8 (Post-8); and after 12 (Post-12) weeks of eccentric training. All participants underwent a 4-week control period immediately before the training program (Baroni et al., 2013b, c; Geremia et al., 2018a, b). No training sessions were held in the evaluation weeks, and a 1-week interval was observed after the last training session and the evaluation session (Geremia et al., 2018b).

### Measurements of Triceps Surae Muscle Architecture

A B-mode ultrasonography system (SSD-4000; Aloka Inc., Tokyo, Japan) with a linear-array probe operating at 32 Hz (UST-5710, 60 mm, 7.5 MHz, depth 6.0 cm, no image filter) was used to determine FL, PA, and MT of GM, GL, and SO. Triceps surae muscle architecture was determined through measurements conducted while the subject was seated with the hip flexed at 85° (0°= hip fully extended), the knee fully extended, and the ankle in the neutral position (foot surface perpendicular to the shank). All volunteers were instructed to not engage in any vigorous physical activity for 48 h before the tests (Baroni et al., 2013b).

**FIGURE 1:**
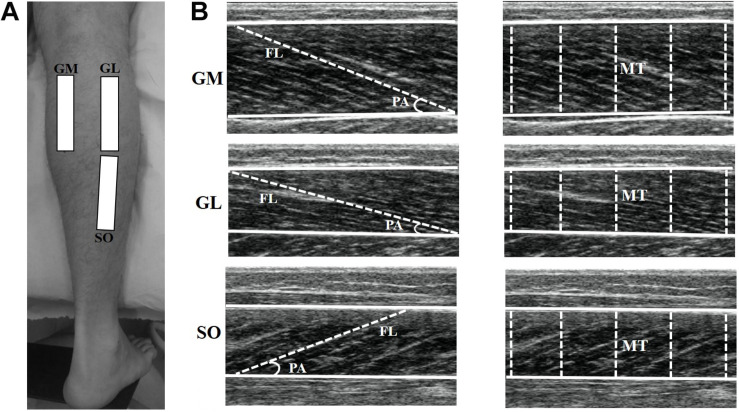
Representation of the ultrasound sites in the gastrocnemius medialis (GM), gastrocnemius lateralis (GL), and soleus (SO) muscles **(A)**. The ultrasonography images show GM, GL, and SO muscles **(B)**. For each muscle, superficial and deep aponeuroses are visualized (continuous lines), among which the fascicle length (FL) and muscle thickness (MT) were measured. Pennation angle (PA) was calculated as the angle between the muscle fascicle and the deep aponeurosis.

Three ultrasound (US) images were obtained for each muscle with the subject at rest. The US probe was covered with water-soluble transmission gel and positioned longitudinally to the muscle fibers and perpendicular to the skin at 50% (SO) and 30% (GM, GL) of the distance between the popliteal crease and the lateral malleolus (Figure 1A; Kawakami et al., 1998; Fouré et al., 2013; Geremia et al., 2018b). Great care was taken to determine the sites where the images were obtained. The US probe was adjusted on the skin surface, parallel to the superficial and deep aponeuroses, and with clarity of the aligned hyperechoic perimysial intramuscular connective tissue (Bohm et al., 2018). Probe alignment was considered appropriate when superficial and deep aponeuroses were parallel, and several fascicles could be easily delineated without interruption across the image (Baroni et al., 2013b). Anatomical reference points, skin marks, and the ultrasonography scanning sites were mapped on a malleable plastic sheet to ensure that repeated scans were taken from the same site.

Ultrasonography images were analyzed by Image J software (straight line, line color: yellow, version 1.48v, National Institutes of Health, Bethesda, MA, United States). The best fascicle (i.e., the fascicle that could be seen in its entirety from its insertion on the deep aponeurosis into the superficial aponeurosis, or to the US probe field-of-view end) in each image was used for FL and PA analysis. FL was considered as the length of the fascicular path between superficial and deep aponeuroses (Figure 1B). When the ends of the fascicles were outside the US image, FL was estimated as recommended in previous studies (Finni et al., 2001; Avela et al., 2004; Abellaneda et al., 2009). PA was calculated as the angle between the muscle fascicle and the deep aponeurosis (Figure 1B). MT was defined as the distance between the deep and superficial aponeuroses, and was calculated through the mean value of five parallel lines drawn at right angles between the superficial and deep aponeuroses along each ultrasonography image (Figure 1B). Mean values were obtained from three US images for each muscle in order to determine FL and PA (i.e., analyses were based on a total of three fascicles per muscle), as well as MT (Baroni et al., 2013b; Geremia et al., 2018b). All measurements taken during the study were performed by the same investigator with extensive experience in ultrasonography, who was blinded to the identity of the participants and time-point at which each ultrasonography image was obtained.

### Training Program

The eccentric training program was conducted for 12 weeks and was the same as that used in previous studies (Geremia et al., 2018a, b). It encompassed three 4-week mesocycles. The first mesocycle had seven training sessions; the second and third mesocycles had eight training sessions each. The smaller number of training sessions in the first mesocycle was chosen to avoid excessive muscle damage in the first training week (Baroni et al., 2013c). Therefore, the training program was comprised of 23 training sessions, and, except for the 1st week, all training sessions were executed twice a week, respecting a 72-h interval between consecutive sessions. Training volume was gradually augmented and consisted of 3 × 10 repetitions in weeks 1 to 4, 4 × 10 repetitions in weeks 5–8, and 5 × 10 repetitions in weeks 9–12.

Before each training session, participants performed a 10-min warm up on a cycle ergometer with a 100 W constant power output (Duclay et al., 2009; Geremia et al., 2018b). Next, a specific warm up (1 × 10 repetitions of submaximal plantarflexion and dorsiflexion concentric contractions at an angular velocity of 120°.s^–1^) was executed on an isokinetic dynamometer (*Biodex System 3 Pro, Biodex Medical Systems, United States*) with the subject seated with the hip flexed at 85° (0°= hip fully extended) and the knee fully extended. The eccentric training started at an ankle angle corresponding to 80% of the maximal dorsiflexion angle (DFmax) and covered a range of 50° (Geremia et al., 2018a, b). DFmax was re-evaluated passively at the isokinetic dynamometer every 4 weeks in order to re-adjust maximal ankle joint range of motion.

During training, participants were instructed to resist the dorsiflexion motion generated by the dynamometer using maximal effort contractions. Participants executed eccentric contractions only during the training program. At the end of each eccentric contraction, the participant’s foot was passively moved back to the initial position for the next eccentric contraction. Participants were encouraged to execute maximal plantar flexion effort as soon as the dynamometer arm reached the initial position. All repetitions were executed continuously, with a 1-min interval between series. Both limbs were trained to avoid inter-limb muscle imbalances. However, only the dominant limb was used for data analysis (18 right limbs and two left limbs). Subjects were asked about the leg they used to kick a ball, which was considered the dominant leg (Geremia et al., 2015).

### Statistics

All statistical analyses were executed in SPSS Statistics software (IBM, version 20, United States) with a 5% significance level (α ≤ 0.05). Results are presented in tables by means ± standard deviations, and in the figures by means ± standard errors.

An intra-class correlation coefficient (ICC) was used to determine test-retest reliability between Baseline and Pre-training values. Reliability was interpreted according to Landis and Koch’s scale (Landis and Koch, 1977) as: <0.00 poor, 0.0–0.2 slight, 0.21–0.4 fair, 0.41–0.6 moderate, 0.61–0.8 substantial, and 0.81–1.0 almost perfect.

A repeated measures one-way ANOVA was used to determine possible training effects as a function of time (i.e., difference between the Baseline, Pre-training, Post-4, Post-8, and Post-12 evaluation time-points) for FL, PA, and MT for each muscle. Normality of the target variables was determined using the Shapiro–Wilk test. The Mauchly test and the Greenhouse–Geisser correction were applied when data sphericity was not obtained. A Bonferroni *post hoc* test was used to identify between time-point differences (Baseline, Pre-training, Post-4, Post-8, and Post-12) for each outcome. ES (Cohen’s *d*) was calculated and classified as trivial (*d* < 0.2), small (*d* > 0.2), moderate (*d* > 0.5), or large (*d* > 0.8) (Cohen, 1988).

Relative changes to the Pre-training evaluation were determined for each outcome variable. A repeated measures two-way ANOVA was used for the between-muscles (GM, GL, and SO) and between-time points (Baseline, Pre-training, Post-4, Post-8, and Post-12) comparisons. When interaction between muscle and time-point was observed, a one-way ANOVA was used at each evaluation time to compare the relative change between the GM, GL and SO muscles, while a one-way repeated measures ANOVA, followed by a Bonferroni *post hoc* test was used in each muscle to compare the different evaluation times.

Responsiveness to the eccentric training (percent change from pre- to post-training) was determined by the typical error (TE) criteria (Cadore et al., 2018). The TE was calculated by the equation TE = SD_diff_/√2, where SD_diff_ is the standard deviation of the differences between the evaluation time-points of Baseline and Pre-training. Non-responsive participants were defined as those that did not achieve an increase that was two times higher than the TE with respect to zero.

## Results

High scores for test-retest reliability between baseline and pre-training evaluations were obtained for all measures. An almost perfect (Landis and Koch, 1977) result was observed for all outcomes: FL (GM = 0.903; GL = 0.914; SO = 0.934), PA (GM = 0.897; GL = 0.884; SO = 0.904), and MT (GM = 0.975; GL = 0.982; SO = 0.979). Baseline and Pre-training values were similar for all outcomes (*p* > 0.05; ES < 0.2; Table 1).

**TABLE 1:** Fascicle length (FL), pennation angle (PA), and muscle thickness (MT) from gastrocnemius medialis (GM), gastrocnemius lateralis (GL), and soleus (SO).

		**Baseline**	**Pre-training**	**Post-4**	**Post-8**	**Post-12**
GM	FL (cm)	5.36^a^±0.65	5.33^a^±0.69	5.81^b^±0.72	6.00^c^±0.73	6.03^c^±0.85
	PA (°)	20.61^a^±2.27	20.35^a^±2.25	20.87^a^±2.34	20.92^a^±2.65	21.22^a^±2.70
	MT (cm)	1.86^a^±0.24	1.85^a^±0.23	1.98^b^±0.22	2.11^c^±0.24	2.12^c^±0.27
GL	FL (cm)	6.32^a^±1.00	6.39^a^±1.03	6.77^b^±1.15	6.89^b^±1.14	6.95^b^±1.18
	PA (°)	12.39^a^±1.32	12.59^a^±1.48	13.37^a^±2.15	13.34^a^±1.52	13.61^a^±2.08
	MT (cm)	1.37^a^±0.17	1.38^a^±0.18	1.53^b^±0.16	1.57^b^±0.15	1.60^b^±0.16
SO	FL (cm)	4.60^a^±0.85	4.62^a^±0.86	5.14^b^±1.07	5.43^c^±1.14	5.57^c^±1.04
	PA (°)	18.29^a^±1.80	18.58^a^±2.00	18.59^a^±3.94	19.39^a^±4.47	18.80^a^±3.60
	MT (cm)	1.55^a^±0.18	1.56^a^±0.18	1.74^b^±0.25	1.85^c^±0.28	1.85^c^±0.23

*All values are mean ± sd. Different letters indicate differences between moments (*p* ≤ 0.05).*

All triceps surae muscles increased their FL in response to eccentric training (GM: *p* < 0.001, ES = 0.90; GL: *p* < 0.001, ES = 0.51; SO: *p* < 0.001, ES = 1.00; Table 1). The three muscles increased their FL in the first four training weeks. GM and SO continued to increase their FL from Post-4 to Post-8, while GL did not. None of the three muscles had FL changes between Post-8 and Post-12 (Table 1). The individual responsiveness analysis showed that 60–90% of the participants responded to eccentric training with FL increases (Table 2).

**TABLE 2:** Individual responsiveness to eccentric training.

		Typical error	Responders *n* (%)	Non-responders *n* (%)
GM	FL	0.21	14 (70)	06 (30)
	PA	0.72	10 (50)	10 (50)
	MT	0.04	18 (90)	02 (10)
GL	FL	0.30	12 (60)	08 (40)
	PA	0.48	08 (40)	12 (60)
	MT	0.02	19 (95)	01 (05)
SO	FL	0.22	18 (90)	02 (10)
	PA	0.59	07 (35)	13 (65)
	MT	0.03	17 (85)	03 (15)

*GM, gastrocnemius medialis; GL, gastrocnemius lateralis; SO, soleus; FL, fascicle length; PA, pennation angle; MT, muscle thickness.*

Pennation angle did not change along the training period for any muscle (*p* > 0.05; ES < 0.2; Table 1). According to the individual responsiveness analysis, 35–50% of the participants presented changes on PA in response to the eccentric training (Table 2).

Muscle thickness increase was consistent among the muscles assessed in this study (GM: *p* < 0.001, ES = 1.08; GL: *p* < 0.001, ES = 1.29; SO: *p* < 0.001, ES = 1.40; Table 1). Just as observed for FL, the three muscles increased their MT in the first four training weeks, and GM and SO continued to increase from Post-4 to Post-8. None of the three muscles had MT changes between Post-8 and Post-12 (Table 1). The individual responsiveness analysis shows that 85–95% of the participants responded to eccentric training with MT increases (Table 2).

**FIGURE 2:**
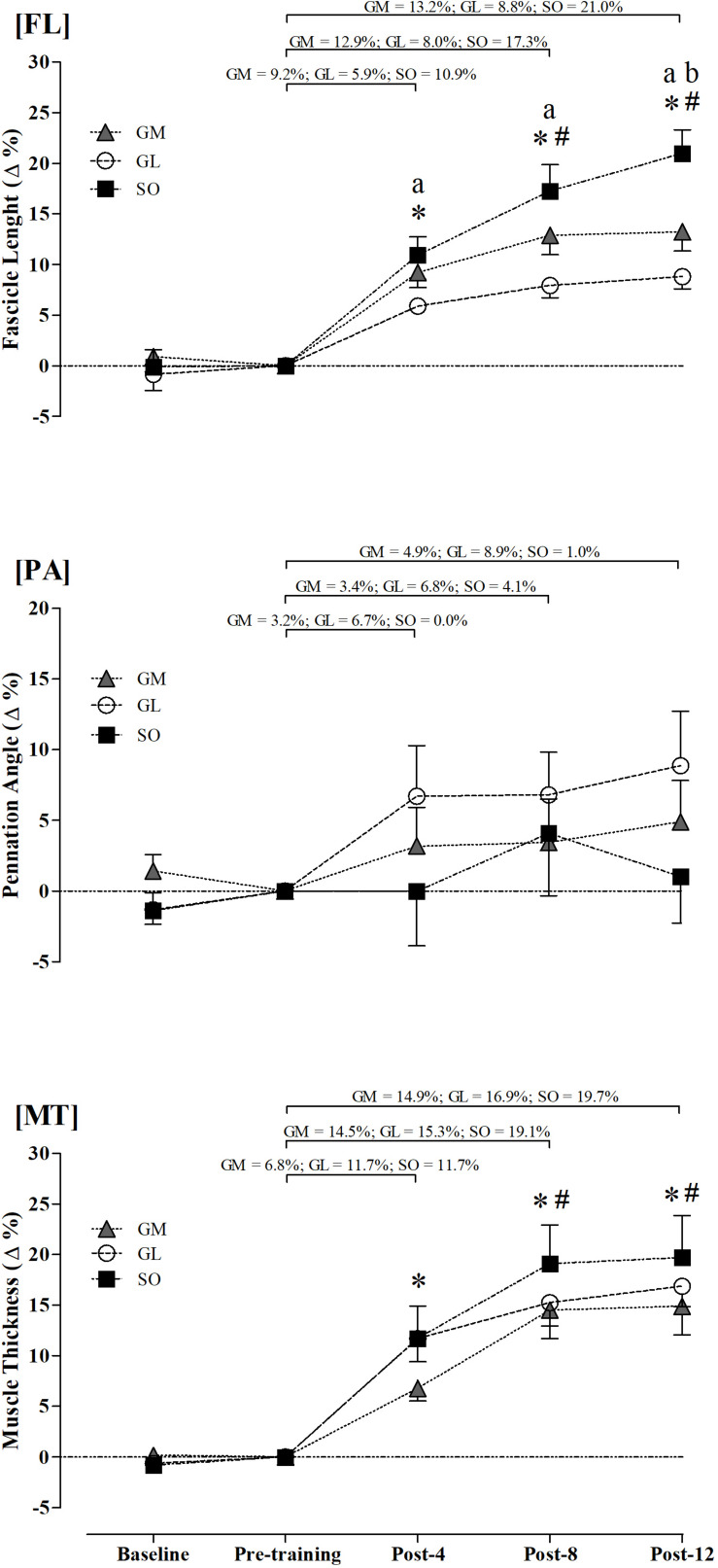
Relative changes in fascicle length (FL), pennation angle (PA), and muscle thickness (MT) of gastrocnemius medialis (GM), gastrocnemius lateralis (GL), and soleus (SO). ^∗^Different from pre-training (*p* ≤ 0.05). ^#^Different from Post-4 in the GM and SO muscles (*p* ≤ 0.05). ^a^Indicates differences between SO and GL muscles (*p* ≤ 0.05). ^b^Indicates differences between SO and GM muscles (*p* ≤ 0.05).

As shown in Figure 2, the percent change analysis further supports the continuous increase in FL and MT up to eight training weeks, with no consistent change in the PA. SO and GM had greater FL percent changes than GL at Post-4, Post-8, and Post-12, while SO had greater values than GM at Post-12 (Figure 2). The three muscles had similar MT percent changes along the training program (Figure 2).

## Discussion

The purpose of this study was to determine the effects of a 12-week eccentric training program on the triceps surae FL, PA, and MT. The primary findings of this study were that: (1) eccentric training leads to an increase in FL and MT, while PA does not change, (2) FL of SO showed the greatest relative increase, and (3) 4 weeks of eccentric training are sufficient to cause architectural changes in all triceps surae muscles.

Fascicle length increase is a commonly found adaptation with eccentric training (Blazevich and Sharp, 2005; Blazevich et al., 2007; Duclay et al., 2009; Baroni et al., 2013b; Timmins et al., 2016b). Studies in animal models (Lynn et al., 1998; Butterfield et al., 2005) support the idea that eccentric training leads to serial sarcomere addition (sarcomerogenesis) and, consequently, increases in FL. This increase in serial sarcomeres might be related to eccentric contraction-induced muscle micro-damage, which activates processes of tissue repair potentially causing serial sarcomere addition in myofibrils (Proske and Morgan, 2001; Baroni et al., 2013b). This serial sarcomere addition increases FLs thereby reducing individual sarcomere lengths for a given muscle length and joint configuration. Since eccentric contraction-induced damage has been shown to critically depend on sarcomere length, increasing sarcomere number and decreasing sarcomere lengths has been thought to be a potent strategy for mitigating eccentric muscle damage (Morgan, 1990; Proske and Morgan, 2001; Brughelli and Cronin, 2007; Lieber, 2018). It has also been argued that increasing serial sarcomere number may increase a muscle’s compliance (Proske and Allen, 2005), thereby minimizing the effects of unstable regions (descending phase) of the force-length relation during eccentric contractions (Proske and Allen, 2005; Brughelli and Cronin, 2007), but the theories of instability on the descending limb of the force-length relationship have largely been shown to be not tenable (Allinger et al., 1996; Rassier et al., 2003). Some characteristics of our eccentric training program may have potentiated the FL increase. Considering that elevated eccentric loads (Reeves et al., 2009) and high intensity stretching (Freitas and Mil-Homens, 2015) lead to FL increase, our elevated loads (3–5 series × 10 maximal repetitions) and the large elongation (80% DFmax) caused by our eccentric training program may have optimized conditions for large serial sarcomere additions, leading to the observed FL increase.

Fascicle length increase is associated with important functional changes, such as increased joint range of motion (Potier et al., 2009; Freitas and Mil-Homens, 2015), and a shift of the length of optimal force production toward longer muscle length (Brughelli and Cronin, 2007). We recently demonstrated, in the same participants, that the eccentric protocol used in this study was associated with an increase in DFmax, and a shift in the plantar flexor torque production optimal length toward longer lengths (Geremia et al., 2018b). Among the advantages of such a shift in optimal muscle length is a reduction in the risk for muscle strains by overstretching (Brockett et al., 2001; Brughelli and Cronin, 2007). Plantar flexor muscle strain injuries occur frequently in sports that demand high running speed or running volumes, with high acceleration and deceleration phases (Green and Pizzari, 2017). It has also been suggested that long fascicle excursion reduces the risk for plantar flexors strain injuries (Green and Pizzari, 2017). Although there is not sufficient evidence for a causal relationship between FL and plantar flexor strain injury, such a relationship has been found for the hamstring muscles (Timmins et al., 2016a). In addition, Ribeiro-Alvares et al. (2018) demonstrated that eccentric training (Nordic hamstring exercise; 4 weeks) reduced risk factors typically associated with the hamstring strain injuries. Therefore, eccentric training might also be an important approach in reducing the risk for plantar flexor strain injuries through an increase in triceps surae FL.

An increase in FL may also have implications for sports performance. Theoretically, long-fibered muscles should be able to generate greater force at high shortening velocities compared to short-fibered muscles, as each sarcomere has a characteristic maximal speed of shortening, and this shortening speed is directly dependent on the number of sarcomeres in series in a muscle fiber (Lieber and Fridén, 2000; Blazevich, 2006). High maximal shortening velocities favor performance in which the shortening velocity is high and power requirements are large. Athletes with long FL have been shown to have better sprint performance than athletes with short fiber length (Kumagai et al., 2000; Abe et al., 2001; Mangine et al., 2014; Nasirzade et al., 2014). Kumagai et al. (2000) found a positive correlation between FL and 100 meters running performance in elite sprinters. Similarly, lower limb FL in sprint runners have been shown to be longer than in non-sprint runners (Abe et al., 2000; Lee and Piazza, 2009). Increased FL might also be an important aspect when developing training programs for the elderly. Aging is associated with neural (Clark and Manini, 2008; Aagaard et al., 2010) and structural (i.e., sarcopenia) (Andersen, 2003; Doherty, 2003) adaptations that reduce a muscle’s force-velocity and power-velocity ability (Vandervoort, 2002; Baroni et al., 2013a). Sarcopenia has been associated with a reduction in FL (Doherty, 2003; Baroni et al., 2013a), and this reduction appears to account for almost half of the difference in muscle shortening velocity between young and elderly subjects (Thom et al., 2007). With that in mind, eccentric training may prove to be an important intervention to prevent sarcopenia, strength and power loss with aging (Reeves et al., 2009).

The SO showed a greater relative increase in FL than GL. This finding might be explained by the different initial FL in these muscles. Previous studies suggested that FL is a crucial determinant for eccentric training-induced muscle damage (Lieber and Friden, 1999; Proske and Morgan, 2001). Short compared to long muscle fibers are thought to be potentially more susceptible to muscle damage as they may work closer to the descending limb of the force-length relationship (Baroni et al., 2013b), causing greater strains and micro-damage in short compared to long muscle fibers (Lieber and Friden, 1999; Proske and Morgan, 2001). Among the triceps surae muscles, the SO has the shortest and GL the longest FL (Wickiewicz et al., 1983; Maganaris et al., 1998; Fouré et al., 2013). Assuming similar moment arms for these muscles, similar excursion afforded by changes in the angle of pennation, and similar compliance in the series elastic elements, this structural difference between SO and GL may lead to greater micro-damage in SO than GL, thereby explaining the greater relative increase in FL in SO compared to GL. However, in order to test this theory properly, the excursions of the muscle fibers during the eccentric protocol would need to be quantified carefully.

In addition to an increase in serial sarcomeres, strength training is typically associated with muscle hypertrophy characterized by an increase in sarcomeres arranged in parallel (Schoenfeld, 2010), which has been used to explain increases in PA of hypertrophied muscles (Kawakami et al., 1993). Although some eccentric training studies reported an increase in PA (Blazevich et al., 2007; Duclay et al., 2009), we did not find such an increase in our study. Our results agree with previous studies (Baroni et al., 2013b; Fouré et al., 2013). The absence of a change in PA post-eccentric training might be due to measurement errors in US image analysis (Baroni et al., 2013b). While eccentric training and conventional resistance training seem to increase plantar flexor PA by around 1.2–4.0° (Morse et al., 2007; Duclay et al., 2009; Vieillevoye et al., 2010; Sanz-Lopez et al., 2016), ultrasonographic analyses have shown a TE of 0.15–3.7 (de Boer et al., 2008; Padhiar et al., 2008; Martins et al., 2012; McMahon et al., 2016). Reported PA adaptive responses to strength training are close to the measurement error in US analysis, which could explain results from studies that found an increase in muscle hypertrophy without concomitant changes in PA (Reeves et al., 2009; Raj et al., 2012; Baroni et al., 2013b; Fouré et al., 2013).

The main limitation of our study is the lack of a control group. However, a 4-week control period was used to verify the outcomes’ reliability and to establish a baseline period for each participant, as previously done in other studies (Izquierdo et al., 2001; Hakkinen et al., 2003; Baroni et al., 2013b, c; Geremia et al., 2018a, b). As shown in Table 2, there were no changes in the outcomes during the control period. In addition, our training program used eccentric isokinetic contractions that allow for a high load and precise velocity control. However, during activities of daily living, external loads are applied with different magnitudes and at different velocities, and therefore our protocol does not resemble what happens in mechanical load exposition in everyday life. Therefore, exercises allowing a larger external load variability (e.g., use of body weight) might be more interesting for clinical practice, and it would be interesting to see if, in rehabilitation programs, the commonly used eccentric exercises do lead to similar adaptations during clinical practice as those here observed. Finally, the high intensity used in our eccentric training program can promote excessive overload on the tendon, which could contribute to the development of tendinopathy (Fredberg and Stengaard-Pedersen, 2008; Bohm et al., 2015). Therefore, although our results indicate that our eccentric training program produced important adaptations at the triceps surae muscle, care should be taken when introducing this or other high intensity eccentric exercise programs aimed at improving physical fitness, injury prevention or injury rehabilitation.

We conclude that 12 weeks of eccentric training increased the triceps surae FL and MT. However, no changes were observed for PA for any of the triceps surae muscles throughout the training program. The SO muscle presented the highest structural adaptations, whereas GL showed the smallest adaptability to eccentric training among the three synergistic muscles.

## Data Availability Statement

The datasets generated for this study are available on request to the corresponding author.

## Ethics Statement

The studies involving human participants were reviewed and approved by the Ethics Research Committee of the Universidade Federal do Rio Grande do Sul (Protocol number: 787.347; CAAE: 32907414.9.0000.5347). The patients/participants provided their written informed consent to participate in this study.

## Author Contributions

JG, BB, and MV contributed to the study conception and design. JG, FL, and AL contributed to the data acquisition. JG, BB, FL, RB, AL, WH, and MV contributed to the analysis and interpretation of data, drafting of manuscript, and critical revision. All authors approved the final version of the manuscript.

## Conflict of Interest

The authors declare that the research was conducted in the absence of any commercial or financial relationships that could be construed as a potential conflict of interest.
