# Corrigendum: Triceps Surae Muscle Architecture Adaptations to Eccentric Training

**DOI:** 10.3389/fphys.2020.00627

**Published:** 2020-07-03

**Authors:** Jeam Marcel Geremia, Bruno Manfredini Baroni, Rodrigo Rico Bini, Fabio Juner Lanferdini, Amanda Rodrigues de Lima, Walter Herzog, Marco Aurélio Vaz

**Affiliations:** ^1^Laboratório de Pesquisa do Exercício, Escola de Educação Física, Fisioterapia e Dança, Universidade Federal do Rio Grande do Sul, Porto Alegre, Brazil; ^2^Departamento de Fisioterapia, Universidade Federal de Ciências da Saúde de Porto Alegre, Porto Alegre, Brazil; ^3^Holsworth Research Initiative, La Trobe Rural Health School, La Trobe University, Bendigo, VIC, Australia; ^4^Laboratório de Biomecânica, Centro de Desportos, Universidade Federal de Santa Catarina, Florianópolis, Brazil; ^5^Faculty of Kinesiology, Engineering, Medicine and Veterinary Medicine, University of Calgary, Calgary, AB, Canada

**Keywords:** eccentric exercise, muscle architecture, muscle plasticity, triceps surae, ultrasonography

In the original article, an incorrect abbreviation for muscle thickness, “ML,” was used instead of “MT.” A correction has been made to the **Abstract**, subsection **Results**:

“Fascicle lengths (GM: 13.2%; GL: 8.8%; SO: 21%) and MT (GM: 14.9%; GL: 15.3%; SO: 19.1%) increased from pre- to post-training, whereas PAs remained similar. GM and SO FL and MT increased up to the 8th training week, whereas GL FL increased up to the 4th week. SO displayed the highest, and GL the smallest gains in FL post-training.”

In addition, the training responsiveness was mistakenly calculated considering the Pre-training and Post-12 moments. Thus, “Pre-training and Post-12” was published instead of “Baseline and Pre-training,” which is the correct calculation. Corrections have been made in the following places:

The **Materials and Methods** section, subsection **Statistics**, the final paragraph:

“Responsiveness to the eccentric training (percent change from pre- to post-training) was determined by the typical error (TE) criteria (Cadore et al., 2018). The TE was calculated by the equation TE = SD_diff_/√2, where SD_diff_ is the standard deviation of the differences between the evaluation time-points of Baseline and Pre-training. Non-responsive participants were defined as those that did not achieve an increase that was two times higher than the TE with respect to zero.”

The **Results** section, paragraphs 2-4:

“All triceps surae muscles increased their FL in response to eccentric training (GM: *p* < 0.001, ES = 0.90; GL: *p* < 0.001, ES = 0.51; SO: *p* < 0.001, ES = 1.00; **Table 1**). The three muscles increased their FL in the first four training weeks. GM and SO continued to increase their FL from Post-4 to Post-8, while GL did not. None of the three muscles had FL changes between Post-8 and Post-12 (**Table 1**). The individual responsiveness analysis showed that 60–90% of the participants responded to eccentric training with FL increases (Table 2).

**Table 2:** Individual responsiveness to eccentric training.

		**Typical error**	**Responders *n* (%)**	**Non-responders *n* (%)**
GM	FL	0.21	14 (70)	06 (30)
	PA	0.72	10 (50)	10 (50)
	MT	0.04	18 (90)	02 (10)
GL	FL	0.30	12 (60)	08 (40)
	PA	0.48	08 (40)	12 (60)
	MT	0.02	19 (95)	01 (05)
SO	FL	0.22	18 (90)	02 (10)
	PA	0.59	07 (35)	13 (65)
	MT	0.03	17 (85)	03 (15)

*GM, gastrocnemius medialis; GL, gastrocnemius lateralis; SO, soleus; FL, fascicle length; PA, pennation angle; MT, muscle thickness*.

Pennation angle did not change along the training period for any muscle (*p* > 0.05; ES < 0.2; **Table 1**). According to the individual responsiveness analysis, 35–50% of the participants presented changes on PA in response to the eccentric training (Table 2).

Muscle thickness increase was consistent among the muscles assessed in this study (GM: *p* < 0.001, ES = 1.08; GL: *p* < 0.001, ES = 1.29; SO: *p* < 0.001, ES = 1.40; **Table 1**). Just as observed for FL, the three muscles increased their MT in the first four training weeks, and GM and SO continued to increase from Post-4 to Post-8. None of the three muscles had MT changes between Post-8 and Post-12 (**Table 1**). The individual responsiveness analysis shows that 85–95% of the participants responded to eccentric training with MT increases (Table 2).”

Finally, as the training responsiveness was miscalculated, Table 2 needs to be corrected. The corrected Table 2 appears above.

The authors apologize for this error and state that this does not change the scientific conclusions of the article in any way. The original article has been updated.
